# Obituary

**Published:** 1874-01

**Authors:** 


### OBITUARY.

### *The late Dr. Milton Parker.*

At a regular meeting of the Chicago Medical Society held on Monday evening, December 15, 1873, the following resolutions were passed :

WHEREAS, It has pleased Divine Providence to remove from our midst, Dr. Milton Parker, after a long life of activity and usefulness—

*Resolved*, That in his death the medical profession has lost a competent and conscientious member, humanity an exemplar, and society an accomplished gentleman.

*Resolved*, That the Chicago Medical Society deeply deplores the bereavement of the family and friends, and extends to them its heartfelt sympathy.

*Resolved*, That a copy of these resolutions be furnished for publication to the medical journals and daily papers of the city, and that the family and friends be informed of the action of the Society.

### *Joseph Woodward—Resolutions of Respect.*

At a meeting of the students of Rush Medical College, held on Thursday, Nov. 6, 1873, to commemorate the death of Joseph Woodward, a member of the Senior Class of 1873–4, the following resolutions were unanimously adopted :

WHEREAS, Death has again painfully reminded us that we are mortal, in the removal of our fellow student, Joseph Woodward, a senior member of the Class of 1873-4, November 5, 1873, and, whereas we desire to give an expression of our sympathy with friends, and offer a tribute of respect to him, therefore—

*Resolved*, That in our deceased classmate, Joseph Woodward, we recognized and admired all those noble qualities which make the true man and the earnest student, and that, in his death, not only we, his classmates, but the future of the profession and the world have met with an irreparable loss.

*Resolved*, That the heartfelt sympathy of the Class of 1873–4 is hereby tendered the bereaved wife and family of the deceased in their great sorrow.

*Resolved*, That a copy of these resolutions be transmitted to the bereaved family, and published in the CHICAGO MEDICAL JOURNAL.

